# The habitual motion path theory: Evidence from cartilage volume reductions in the knee joint after 75 minutes of running

**DOI:** 10.1038/s41598-020-58352-5

**Published:** 2020-01-28

**Authors:** Steffen Willwacher, Daniela Mählich, Matthieu B. Trudeau, Joseph Hamill, Gillian Weir, Gert-Peter Brüggemann, Grischa Bratke

**Affiliations:** 1 Institute of Biomechanics and Orthopaedics, German Sport University, Cologne, Germany; 2 Brooks Sports Inc., Seattle, Washington, USA; 3 Biomechanics Laboratory, University of Massachusetts, Amherst, MA USA; 4 Department of Diagnostic and Interventional Radiology, University of Cologne, Cologne, Germany

**Keywords:** Biophysics, Cartilage

## Abstract

The habitual motion path theory predicts that humans tend to maintain their habitual motion path (HMP) during locomotion. The HMP is the path of least resistance of the joints defined by an individual’s musculoskeletal anatomy and passive tissue properties. Here we tested whether participants with higher HMP deviation and whether using footwear that increases HMP deviation during running show higher reductions of knee joint articular cartilage volume after 75 minutes of running. We quantified knee joint articular cartilage volumes before and after the run using a 3.0-Tesla MRI. We performed a 3D movement analysis of runners in order to quantify their HMP from a two-legged squat motion and the deviation from the HMP when running in different footwear conditions. We found significantly more cartilage volume reductions in the medial knee compartment and patella for participants with higher HMP deviation. We also found higher cartilage volume reductions on the medial tibia when runners wore a shoe that maximized their HMP deviation compared with the shoe that minmized their HMP deviation. Runners might benefit from reducing their HMP deviation and from selecting footwear by quantifying HMP deviation in order to minimize joint cartilage loading in sub-areas of the knee.

## Introduction

Overuse injuries in distance running occur with gradual onset over time and result from the repetitive stress of biological tissues and associated cumulative trauma^1^. The knee joint is the most common site for running-related overuse injuries (RROIs)^2^. Nigg *et al*. have proposed that the neural control of running is tuned towards minimizing mechanical stress of biological tissues, resulting in an optimal path of lower extremity joint movement for each individual and every specific movement^3^. Recently, we proposed a redefinition of this theory by assuming that the neural control of running is tuned towards keeping the individual’s habitual joint motion path (HMP), which we define as the joints’ path of least resistance, and a function of an individual’s anatomy and passive tissue mechanical properties^4^. As such, the HMP is the set of joint kinematics during motions which minimizes loading of lower extremity joints relative to the loads during running. Examples for these relatively low loading motions are walking, stair climbing, sitting down or standing up from a chair. Consequently, we have developed a simple method to estimate the HMP for individuals from a basic half-squat movement^4^.

Running at typical distance running speeds requires greater force application to the ground in order to satisfy the body weight support requirement during shorter ground contact times compared to walking^5,6^. Therefore, runners must generate amplified lower extremity joint moments when running in order to maintain the HMP and to optimize the load distribution to regions that have been adapted to carry these loads. Based on the HMP theory, deviating from the HMP leads to loading of less adapted structures of lower extremity joints, resulting in a greater risk of sustaining a running-related overuse injury. From the same theoretical background, we proposed that running footwear selection should account for the minimization of the deviation from the HMP^4^. Footwear that does not fulfill this task would lead to deviation from the HMP or additional muscle activity to keep the HMP. Both of these consequences would result in greater loading of joint structures.

The HMP theory was derived from the assumption that the neural control of running is adjusted to avoid irreversible injuries such as osteoarthritis. These optimization strategies might have also been essential for human evolution^7^. While the HMP theory was developed from a sound theoretical background, it has only very rarely been tested empirically^4,8–10^.

Measuring the loads imposed on cartilage structures in a running participant non-invasively *in-vivo* is not feasible using current technology. Nonetheless, with high-resolution magnetic resonance imaging (MRI) one can measure cartilage volumes before and after a prolonged run^11–14^. Cartilage volume may act as a surrogate variable to indirectly quantify the loading imposed onto the cartilage^15^. Knee joint cartilage morphology has been related to biomechanical loading characteristics of the knee during locomotion^15^.

Therefore, the purpose of the present study was to test hypotheses derived from the HMP theory. Specifically, we hypothesized that knee joint cartilage loading, quantified via cartilage volume reductions after 75 minutes of running, would be lower in runners who maintained a lower HMP deviation during running compared to runners who deviated more from their HMP. Further, we hypothesized that specific footwear that minimized HMP deviation in a runner would also minimize cartilage volume reductions during prolonged running. Due to the high prevalence of RROI at the knee^2^, we focused on the knee joint in this study.

## Methods

### Subjects and materials

We recruited twelve participants (seven males and five females; height: 1.77 ± 0.08 m; body mass: 70.9 ± 9.9 kg; age: 29 ± 4 years) for this study which involved a unique multi-visit MRI-based protocol. All participants were recreational runners and were injury free for at least one year before the study. None of the participants had known injuries of the articular cartilage of the knee joint. The ethics committee of the University of Cologne, Cologne, Germany had approved the study protocol. All procedures were carried out in compliance with the declaration of Helsinki. We obtained written informed consent from all participants.

We performed data collection sessions during 4 different days, both in a biomechanics laboratory (1 visit) and in the MRI facilities of the University hospital (3 visits; at least one week between individual visits).

### Running mechanics and habitual motion path determination

In the biomechanics laboratory, we analyzed the 3D running kinematics and kinetics of the participants while running on a 3D force instrumented treadmill (Treadmetrix, Park City, UT, USA) at the participants’ self-selected speed using three different footwear conditions. The participants selected the running speed based on experience such that they would feel comfortable keeping the speed for 75 minutes.

**Figure 1:**
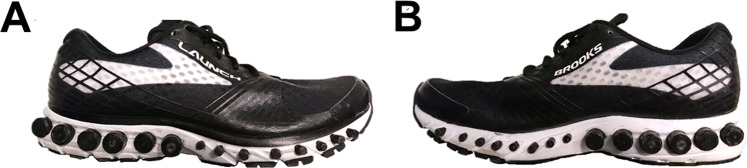
Experimental shoe conditions used in this study. We created a laterally posted condition by inserting plastic tubes in the lateral border of the midsole (**A**) and a medially posted condition by inserting plastic tubes along the medial border of the midsole. (**B**) The neutral shoe was a neutral running shoe (either Brooks Launch or Brooks Glycerin) without any holes or tubes within the midsole (not shown).

The three footwear conditions were: (1) a neutral running shoe (either Brooks Launch or Brooks Glycerin, based on individual preference) with a homogeneous density ethyl vinyl acetate (EVA) midsole (Control) without any modifications; (2) a Brooks Launch shoe modified by inserting stiff plastic tubes along the lateral part in the midsole (shoe A); and (3) a Brooks Launch shoe modified by inserting stiff plastic tubes along the medial part in the midsole (shoe B) (see Fig. 1). We created the custom footwear conditions 2 and 3 using the same baseline shoe; i.e., when hardening one side of the midsole by inserting plastic tubes, the holes on the other side were left empty which effectively reduced the midsole hardness on that side. This way, a medial post was created when inserting tubes on the medial side and vice versa.

We measured runner kinematics using a twelve-camera optoelectronic 3D motion capture system (MX40, Vicon Motion Systems, Oxford, UK). We used a five-segment rigid body model of the pelvis and the right lower extremity to determine 3D joint kinematics. The details of the model can be found in previous publications^16–18^.

During the same lab visit, the subjects performed ten two leg half-squat movements in sock shoes, which consisted in running socks that have been glued to standard sock liners made out of 5.5 mm EVA foam^4,19^, at self-selected speed. The participants placed their feet hip width apart with their feet pointing forwards. From these movements, we estimated the knee joint HMP of each participant while following our recently published protocol^19^. In brief, this protocol quantifies the frontal and transverse plane knee joint angles at a knee flexion angle of 40 degrees during the eccentric phase of the half-squat. With this approach there was only one HMP baseline to compare against running in the different footwear conditions. Please refer to the Supplementary Materials (Supplementary Fig. 1) for a graphical illustration of the deviation quantification. After determining the HMP baseline from the half-squat, the protocol determined these angles at the same critical flexion angle during the eccentric part of the contact phase in the running movement. By subtracting the knee non-sagittal plane angles during the squat baseline from the knee angles obtained in the running movement, the HMP deviation was quantified. Since transverse and frontal plane ranges of motion at the knee joint in running are different, we determined the overall HMP deviation by using a weighted sum of frontal and transverse plane deviations using the following formula:

1 $${{\rm{Dev}}}_{{\rm{HMP}}-{\rm{Tot}}}={{\rm{Dev}}}_{{\rm{HMP}}-{\rm{Front}}}+0.5\cdot {{\rm{Dev}}}_{{\rm{HMP}}-{\rm{Trans}}},$$

where Dev_HMP-Tot_ is the total HMP deviation in the running movement, Dev_HMP-Front_ is the HMP deviation within the frontal plane and Dev_HMP-Trans_ is the deviation within the transverse plane of motion.

### MRI data collection during prolonged running

In the MRI facilities, we installed a treadmill directly in front of the MR room in order to minimize the time between running and MRI measurements, and additional loading to the knee. On different days, each participant ran 75 minutes at the same speed as during the biomechanical assessment in three different footwear conditions. The individual running sessions were completed in randomized order within a time period of less than four weeks. Measurements were taken at the same time of the day for all participants.

We performed knee MRI scans before (pre-run) and immediately after (post-run) each running bout. Furthermore, we performed additional MRI scans of the calf muscles at 2.5, 5, 10, 15 and 45 minutes, which were not related to the purposes of this study. These scans interrupted the treadmill running by 3:40 minutes on average. All participants completed a 30-minute rest phase before the pre-run scan which involved lying on the MR table outside of the MRI scanner so that the participitants could be placed within the scanner without further movement or loading of the knee. We took the pre-run MRI scan for the segmentation after the acquisition of the survey and anatomical proton-density weighted images which lasted another 15 minutes, which meant an overall rest of 45 minutes before the pre-run scan Each participant entered the MRI scanner within ten seconds following the 75-minute run. The start of the scan occurred less than a minute after finishing the run. Given the short time between the end of the run and the completion of the scan, we believe that the subjects’ cartilage recovery was minimal, and therefore that we were able to capture effects from the prolonged run.

We performed the multiple-slice MRIs on a 3.0 Tesla scanner (Ingenia, Philips N.V., Amsterdam, Netherlands) with a 16-channel high resolution transmitting and receiving knee coil. We carefully positioned the participants in a supine orientation while centering the knee joint center within the magnet and attached with the knee coil. We used a 3D water selective (WATS) T1 gradient echo sequence (repetition time: 11 ms; echo time: 5.6 ms; flip angle: 10°) with a field of view of 160 × 160 × 90 mm and 180 partially overlapping slices (gap: −0.5 mm). The size for the acquired voxels was set to 0.493 × 0.493 × 1 mm and 0.286 × 0.286 × 0.5 mm for the reconstruction voxels. The effective scan duration was 5:27 minutes for the complete sequence while using parallel imaging for faster image acquisition (SENSE factor 2).

**Figure 2:**
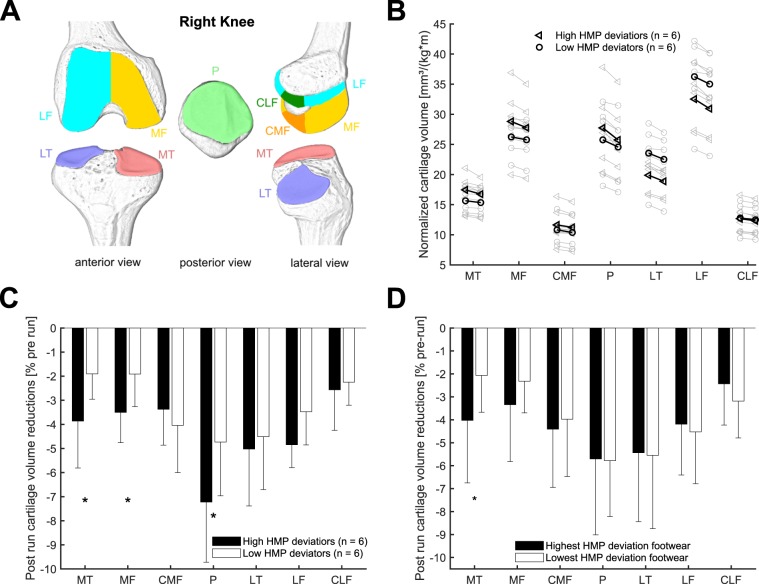
(**A**) Schematic drawing of cartilage sub-areas of interest in this study. (**B**) Individual response in cartilage volumes to the 75-minutes running intervention. Individual data points are the average of the three footwear conditions. Bold data points/lines indicate the mean values of the high and low deviation groups. In each column the left value is the pre-run cartilage volume and the right value is the post-run cartilage volume. (**C**) Relative post-run cartilage volume reductions between post-run and pre-run measurements (means – standard deviations) between runners with high HMP deviation and runners with low HMP deviation in the different knee joint sub-areas. * indicates a significantly higher cartilage volume reduction in the High HMP deviation group (p < 0.05). (**D**) Relative post-run cartilage volume reductions compared to pre-run (means – standard deviations) between the footwear conditions with the highest and lowest overall HMP deviation. *Indicates a significant difference between the two footwear conditions at a level of p < 0.05.

We conducted segmentation of the total subchondral bone area (Piscoya *et al*., 2005) and the cartilage joint surface area (AC) by manual segmentation on a section by section basis with a B-spline Snake algorithm (deformable contour) using custom software (Chondrometrics GmbH, Ainring, Germany). We divided the knee joint into seven anatomical regions^14,20^ (Fig. 2A): patella (P), medial tibial compartment (MT), lateral tibial compartment (LT), the medial (MF) and lateral (LF) femoral condyles, and medial and lateral central (weight bearing area) femoral condyles (CMF and CLF). In these anatomical regions, we calculated the cartilage volume (VC) from three-dimensional reconstructions. We normalized cartilage volumes to body mass and height, and quantified differences between pre and post run cartilage volumes. We expressed these cartilage volume reductions as percentage differences relative to the pre-run condition.

### Statistical analysis

We applied one-tailed Wilcoxon rank sum tests to determine whether the relative cartilage volume reductions of the runners with the highest overall HMP deviation (n = 6) were higher than those of the runners with the lowest HMP deviation (n = 6). To improve the robustness of this analysis, we averaged cartilage volume reductions and HMP deviation values over all three footwear conditions. We further performed comparisons between the footwear conditions with the highest and the lowest individual HMP deviation values using dependent sample t-tests. In addition to these pairwise comparisons, we performed simple linear regression analyses to identify potential relationships between HMP deviation and cartilage volume reductions. We set the level of significance for all tests to 0.05. We quantified effect sizes for between group and between footwear comparisons (Cohen’s d,^21^) using

$$d=\frac{{\bar{x}}_{j}-{\bar{x}}_{i}}{\sqrt{\frac{{s}_{j}^{2}+{s}_{i}^{2}}{2}}},$$

with ${\bar{x}}_{i,j}$ being the average and ${s}_{i,j}^{2}$ being the sample variance of the group or footwear data. Effect sizes of ≥0.2 were considered as small, ≥0.5 as medium, and ≥0.8 as large^21^.

All statistical analyses were performed using Matlab software (R2018b, Statistics and Machine Learning Toolbox, The Mathworks, Natick, MA, USA).

## Results

The participants ran the 75-minute running trials at a constant speed of 2.78 ± 0.38 m/s. The fastest individual performed the running trials at 3.33 m/s and the slowest at 2.31 m/s. We observed significant reductions in cartilage volume in all knee joint sub areas after the 75-minute run compared to the pre-run condition (Fig. 2B–D).

Runners with greater overall HMP deviation values (n = 6, average over all footwear conditions: 12.5 ± 2.7°) were characterized by greater cartilage volume losses in the MT (p = 0.047), MF (p = 0.033) and P (0.033) sub-areas of the knee joint cartilage compared to runners with lower HMP deviation values (n = 6, average over all footwear conditions: 6.2 ± 2.3°)(Fig. 2B,C). There were no significant differences between HMP deviation groups with respect to body mass (p = 0.94; high deviators: 69.7 ± 8.6 kg, low deviators: 70.2 ± 14.9 kg) and body height (p = 0.85; high deviators: 1.77 ± 0.07 m, low deviators: 1.78 ± 0.13 m). We did not find a systematic difference in self-reported physical activity levels between high and low HMP deviator groups. Further, we found no significant differences in running speed between the two groups of runners (p = 0.38; high deviators: 2.71 ± 0.41 m/s, low deviators: 2.93 ± 0.42 m/s).

We identified a significant relationship between overall HMP deviation and the amplitude of MT (R² = 0.43; p = 0.031) and MF (R² = 0.35; p = 0.045) sub area cartilage volume reductions.

For each participant, we determined the shoe with the greatest overall HMP deviation and the shoe with the least HMP deviation. Three, three and six runners had the greatest HMP deviation in the laterally posted, medially posted and neutral shoe conditions, respectively. Two, seven and three runners had the least deviation in the laterally posted, medially posted and neutral shoe conditions, respectively.

**Figure 3:**
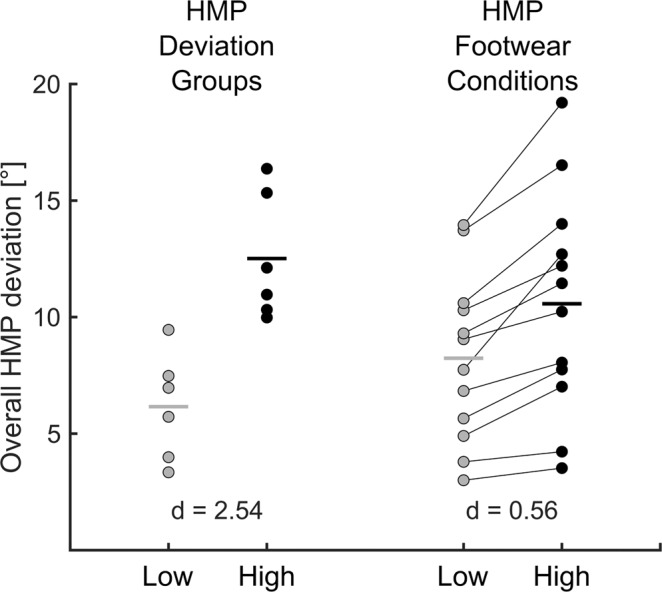
Comparison of HMP deviation amplitudes between the high and low HMP deviation groups (left) and the high and low HMP deviation footwear conditions (right). Each dot on the left part of the graph represents a different runner, while each runner is represented by a different line in the right part of the graph. Bold horizontal lines indicate the mean value of a group of runners (left part) or the mean value of the high and low HMP deviation footwear conditions, respectively (right part). In the bottom part effects sizes (Cohen’s d) for between group and between footwear comparisons are highlighted.

Within the footwear conditions associated with the least overall HMP deviation (8.2 ± 5.9°; Fig. 3) compared to the shoes with the highest HMP deviation (10.6 ± 4.7°; Fig. 3), we found significantly lower cartilage volume reductions in the MT sub-area (p = 0.004, Fig. 2D). We identified a strong linear relationship between the difference between the two extreme footwear conditions in frontal plane deviation and the difference between these shoes in cartilage volume reductions in the CMF sub-area (R² = 0.58; p = 0.004).

The differences in overall HMP deviation between the high and low HMP deviation groups were greater than the differences within subjects when comparing the highest and lowest deviation footwear condition (Fig. 3).

## Discussion

The purpose of the present study was to test hypotheses derived from the HMP theory using cartilage volume reductions as a variable for the indirect quantification of knee joint cartilage loading. We found greater cartilage volume reductions in the medial compartment of the knee in runners with greater deviation from their HMP baseline compared to runners with lower deviation from their HMP baseline. Therefore, we accept our first hypothesis. Furthermore, we found significantly less cartilage volume reduction in the MT sub-area when runners wore footwear with the smallest amount of HMP deviation compared to footwear with the highest amount of HMP deviation. Therefore, we also accept our second hypothesis as it relates to the MT sub-area of the knee.

Cartilage volumes of the femur, tibia and patella cartilage in our study were consistent with previous literature (e.g.^11^) and indicated a high variability of cartilage volume of the analyzed sample (Fig. 2B). Previous studies also reported reduced cartilage volumes after running exercise of different durations and distances^11–13,22^. Boocock *et al*.^11^ identified a reduction of lateral tibial (5.7%), medial femoral (5.3%) and lateral femoral (4.0%) cartilage volumes after a 30-minute running trial. After a 5 km run, Kessler *et al*.^22^ reported a decrease in the patella (6.6%) and tibial (3.6%) cartilage volumes. In general, the cartilage volume reductions reported in this study after the 75-minute running bout agree with previous literature and 30-minutes loading scenarios^11–13,22^.

The results of our study indicate that deviating from the habitual motion path was associated with increased cartilage volume loss in some regions of the knee joint during a prolonged run. This suggests that deviating from the habitual motion path may lead to greater loading on some regions of the knee joint. The greater cartilage volume reductions in runners with greater HMP deviation might have occured because these runners might have loaded cartilage sub-regions which may have been less adapted to mechanical loads given that, habitually, they may be less loaded than other regions of the knee. Loading less adapted cartilage areas has been considered to be a risk factor for the progression of overuse injuries such as knee osteoarthritis^23^. Consequently, monitoring the habitual motion path during well controllable weight-bearing activities such as walking, stair climbing, squatting or sitting down or up from a chair might be an interesting approach to prevent the fast progression of overuse type injuries at the knee.

Furthermore, the development and selection of technological aids such as footwear should consider the HMP of their users. The reduced cartilage volume reduction on the medial tibia across footwear conditions found in this study support this idea, even though the effect sizes induced across footwear were smaller than the effect sizes observed between runners of different HMP deviation patterns. However, the footwear conditions in this study might not have been ideal for minimizing or maximizing HMP deviation for individual participants. It is interesting to note that different shoe conditions minimized or maximized the HMP deviation in individual runners. This highlights the idea that an individual approach is needed to inform footwear assignment for runners which is based on the HMP theory. Future studies need to explore the effects of technological interventions including footwear in greater detail, which might result in a better understanding of the determinants of HMP deviation in running or other more demanding types of locomotion.

We determined the HMP basleline from a two-leg half squat motion as described in our previous protocol paper^4^. We used this motion, because it is a common every day task, similar to e.g. sitting down on a chair. Further, it requires relatively small force generation and can be well controlled by the participants. Therefore, we believe that it is likely that the HMP can be kept by the participants during the two-leg squat. Furthermore, pilot studies inidicated a good reliability of the two-leg squat motion, e.g. in comparison to a one-leg squat motion, which might have the benefit of being more similar to the actual running motion. Future studies should attempt to find a method to quantify the individual HMP which includes several common knee-flexion tasks which likely can be performed while staying within the HMP. This way, a more general and more robust quantification of the HMP baseline might be obtained.

While this study provides new evidence in support of the HMP theory, several limitations need to be considered when interpreting the results. Due to the extensive cost of MRI measurements used in this study, we were only able to include twelve participants in the study. This low sample size was a limiting factor concerning the statistical power of the study. Despite the low sample size, we found several significant differences between subject groups and footwear conditions. However, future studies should try to replicate our findings with a larger sample size in order to further understand the effects of personalized footwear. Next, we used treadmill running to induce running specific loading of the knee joint articular cartilage. The major amount of distance running is performed over ground. Therefore, replication of our results should also consider the use of over ground running, even though the running conditions are much more difficult to standardize in this situation.

Furthermore, we only collected biomechanical data of runners in an non-fatigued state. During a 75 minutes running bout, running mechanics can change because of running-induced fatigue^17,24^. Therefore, future studies should consider fatigue effects when quantifying HMP deviation during prolonged runs.

Finally, measuring knee joint kinematics in the frontal and transverse plane from skin mounted markers is a challenging tasks and is prone to measurement errors, like e.g. soft tissue, skin movement or knee cross-talk artefacts^25–27^. In particular, the soft tissue artefact is dependent upon the muscle activation level and impact characteristics of the movement. Even though we used a mathematical optimization of marker trajectory data in our calculations of the orientation and position of the anatomical coordinate systems, such that the coordinates better comply with rigid body assumptions^10,28^, of the thigh and shank. However, it is not unlikely that some of the HMP deviations calculated were actually caused by measurement errors and not related to actual bone motion differences. Future studies should address this issue by using more precise measurement technologies like e.g. biplanar videoradiography^29^.

In summary, we found evidence in support of the HMP theory. Runners with a higher deviation from the HMP baseline showed significantly greater cartilage volume reductions in several knee joint cartilage sub-regions. Furthermore, the cartilage on the medial tibial compartment showed greater cartilage volume reductions when participants were running in footwear that induced higher HMP deviation. These results indirectly indicate higher mechanical loading on potentially less adapted sub-regions of the knee joint cartilage when runners deviate more from their HMP baseline.

## Supplementary information

Supplemtary Materials.

## Data Availability

All underlying data is made available upon request by the corresponding author of this study.
