# Supplementary material for: The habitual motion path theory: Evidence from cartilage volume reductions in the knee joint after 75 minutes of running
Source: Sci Rep. 2020 Jan 28;10:1363. doi: 10.1038/s41598-020-58352-5 (PMC6987217; doi:10.1038/s41598-020-58352-5)
Supplement: Supplementary file 1 — Supplemtary Materials. [file 41598_2020_58352_MOESM1_ESM.pdf]

Supplementary Materials

The habitual motion path theory: Evidence from cartilage volume reductions in the knee joint after 75 minutes of running

Steffen Willwacher[a*], Daniela Mählich[a], Matthieu B. Trudeau[b], Joseph Hamill[c], Gillian Weir[c], Gert-Peter Brüggemann[a] and Grischa Bratke[d]

[a]Institute of Biomechanics and Orthopaedics, German Sport University, Cologne, Germany
[b] Brooks Sports Inc., Seattle, Washington, USA.
[c]Biomechanics Laboratory, University of Massachusetts, Amherst, MA, USA
[d]Department of Diagnostic and Interventional Radiology, University of Cologne, Cologne, Germany

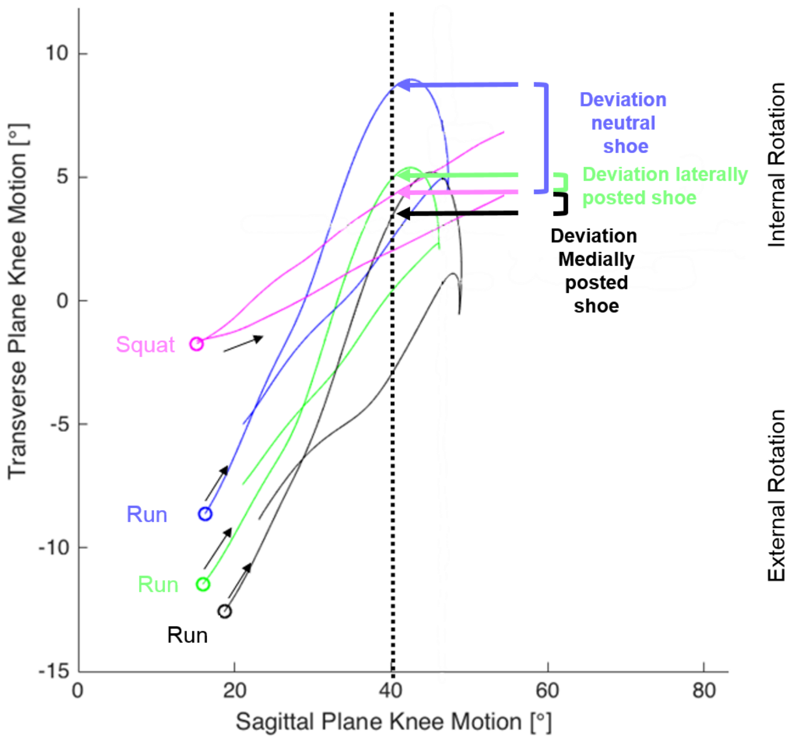

Supplementary Figure 1: Example of the determination of the deviation from the Habitual Motion Path (HMP) baseline in the transverse plane while running in three different running shoes (neutral, medially and laterally posted, respectively). Transverse plane angles at 40° knee flexion for the squat HMP baseline motion and the running motion are indicated by horizontal arrows. The deviation (difference to squat) for each running condition is highlighted by the colored brackets. The transverse plane angles at 40° are taken during the eccentric phase of the knee flexion-extension cycle. Small circles and small black arrows indicating the start of each flexion-extension cycle. For squatting, higher knee flexion angle data was truncated for visualization purposes.
